# Internal mammary sentinel lymph node biopsy with modified injection technique: A case report

**DOI:** 10.1097/MD.0000000000009466

**Published:** 2017-12-29

**Authors:** Zhao Bi, Wei-Zhen Zheng, Heng Qiu, Peng Chen, Peng-Fei Qiu, Yong-Sheng Wang

**Affiliations:** a School of Medicine and Life Sciences, University of Jinan, Shandong Academy of Medical Sciences; b Shandong Cancer Hospital and Institute, Jinan, Shandong, People's Republic of China.

**Keywords:** breast cancer, case report, internal mammary sentinel lymph node, modified injection technique

## Abstract

**Rationale:**

In addition to axillary lymph node (ALN), internal mammary lymph node (IMLN) is also the first-echelon drainage nodes reached by metastasising cancer cells from breast cancer, which can provide important prognostic information.

**Patient concerns:**

In this paper, we will introduce a case of breast cancer patient whose postoperative pathology result showed that she had internal mammary sentinel lymph node (IMSLN) metastases but no axillary sentinel lymph node (ASLN) metastases.

**Diagnoses:**

She was diagnosed as pT1cN1bM0 breast cancer based on the positive IMSLN but negative ASLN.

**Interventions:**

She received axillary-sentinel lymph node biopsy (A-SLNB) and internal mammary-sentinel lymph node biopsy (IM-SLNB) guided by modified injection technique. In the choice of chemotherapy, she received dose-dense AC × 4 times followed *P* × 4 times for chemotherapy. As to irradiation therapy, she received irradiation therapy include chest wall, superclavicular region, and internal mammary nodes.

**Outcomes:**

After performing IM-SLNB, the nodal staging of this patient increased (from N0 to N1b). And she received additional chemotherapy and irradiation therapy.

**Lessons:**

With the guidance of modified injection technique, the preoperative visualization rate of IMLN has been improved. IM-SLNB could be a minimally invasive technique for effective evaluation of the status of IMLN to provide information for staging and guide the adjuvant treatment.

## 1 Introduction

The rate of internal mammary lymph node (IMLN) metastases was reported to be around 18% to 33% (mean 23.4%), with 2% to 11% of patients whose lymph nodes metastases situated only in IMLN without axillary lymph node (ALN).^[1]^ For breast cancer patients, IMLN metastases has a similar prognostic effect as ALN, which is also important for regional staging and subsequent treatment.^[2]^ Axillary-sentinel lymph node biopsy (A-SLNB) has become the standard treatment for patients without clinical and iconography evidence of ALN metastases. But due to the low identification rate of internal mammary sentinel lymph node (IMSLN), internal mammary-sentinel lymph node biopsy (IM-SLNB) is rarely performed clinically. With the appearance of modified injection technique, the identification rate of IMSLN has been significantly improved. IM-SLNB might become a minimally invasive technique to evaluate the status of IMSLN effectively as well as to guide the individualized diagnosis/treatment.^[3]^ One female patient treated in our hospital in August 2014, and she received both A-SLNB and IM-SLNB guided by modified injection technique, whose routine pathology result indicated negative axillary sentinel lymph node (ASLN) but positive IMSLN. Here is the introduction of the case.

## 2 Case presentation

**Figure 1:**
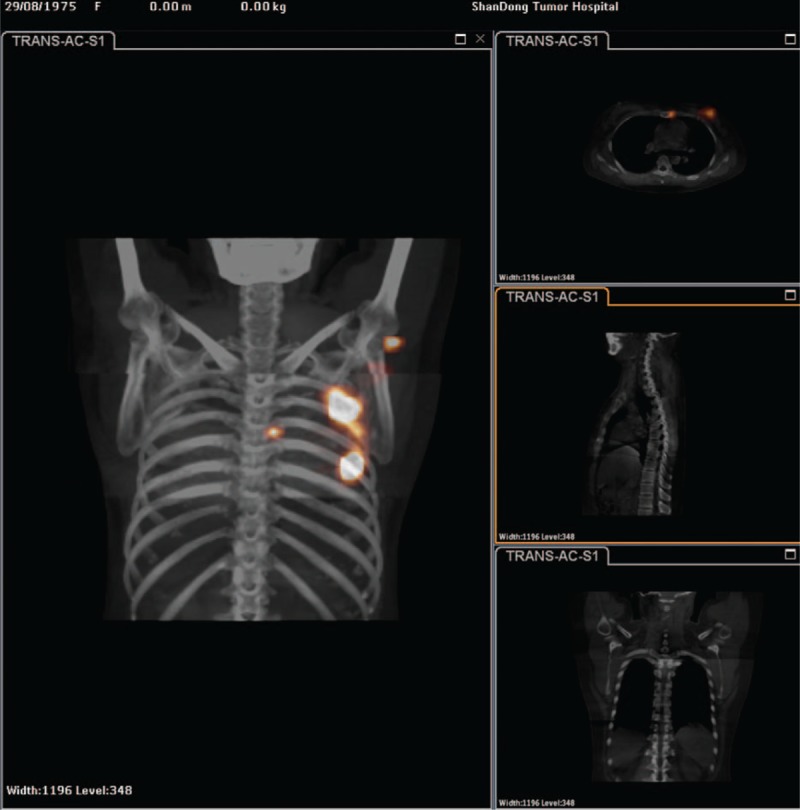
Preoperative lymphoscintigraphy revealed that there was a “hot-spot” in third intercostal space.

Our case was a 48-year-old woman with a painless tumor about 1.5 × 1.0 cm in her left breast for 4 months. She was admitted to our hospital in August, 2014. There were no enlarged ALNs on palpation or ultrasonography. Pathology result of preoperative core needle biopsy confirmed invasive ductal carcinoma. Imaging examination found no metastases in bone or liver. The clinical stage for this patient was cT1cN0M0, IA. She received total mastectomy and A-SLNB and IM-SLNB on August 29, 2014. Under the guidance of ultrasound, 37 MBq of ^99m^Tc-labeled sulfur colloid (^99m^Tc-SC) (1.2 mL volume) was injected into the mammary gland at 6 and 12 o’clock of the areola surrounding area (modified injection technique) 15 hours before surgery. Preoperative lymphoscintigraphy revealed that there was a “hot-spot” in third intercostal space (Fig. 1). Blue dye (4 mL) was injected subcutaneously around the tumor 10 minutes before surgery. Three ASLNs were found with blue dye combined with ^99m^Tc-SC. And the last ASLN was found with ^99m^Tc-SC only. Intraoperative rapid frozen section pathology and touch imprint cytology showed that all of them were negative. After total mastectomy, the first IMSLN was found by the hand-held gamma probe in the third intercostal space as the lymphoscintigraphy revealed. Then IM-SLNB was performed using the mastectomy incision. From the position where IMSLN was located, intercostal muscle fibers were cut off to expose the intercostal space. Then IMSLN was removed and the procedure lasted 10 minutes. In the second intercostal space, we found another IMSLN using gamma probe. This IMSLN was removed in the same way and the procedure lasted only 4 minutes. Both of them are located outside the internal mammary blood vessel. The first IMSLN was about 5 mm in diameter and nuclide count was 10, it did contain metastases after routine pathology. The second IMSLN was about 3 mm in diameter, and the nuclide count was 2, it was negative in the end. The routine pathology indicated: (left breast) invasive ductal carcinoma (1.5 × 1.2 cm), grade II, ASLN (0/4), IMSLN (1/2). The immune-histochemical (IHC) finding presented: ER (−), PR (−) and HER-2 negative (1+), the Ki-67 labeling index was about 60% to 70%. The final pathological stage for this patient was pT1cN1bM0, II A. After performing IM-SLNB, nodal staging of this patient increased (from N0 to N1b). According to the 2017 National Comprehensive Cancer Network (NCCN) Guidelines,^[4]^ this patient received dose-dense AC (Pharmorubicin 100 mg/m^2^ d1 q21d + Cyclophosphamide 600 mg/m^2^ d1 q21d) ×4 times followed by P (Paclitaxel 175 mg/m^2^ d1 q14d) × 4 times for chemotherapy. If IM-SLNB had not been conducted, it is adequate to use TC (Docetaxel 75 mg/m^2^ d1 q21d + Cyclophosphamide 600 mg/m^2^ d1 q21d) × 4 times for chemotherapy in such a low-risk patient. In the choice of irradiation, she finally received irradiation therapy include chest wall, superclavicular region and internal mammary nodes. It is important to note that IM-SLNB has changed adjuvant chemotherapy and irradiation strategy.

The study was approved by the Shandong Cancer Hospital Affiliated to Shandong University Ethics Committee. Informed consent was obtained from this patient for the publication of this case report.

## 3 Discussion

IMLN and ALN belong to the “first station” lymph node of breast cancer lymphatic drainage, which is one of the most important metastatic pathways of breast cancer.^[5]^ There are 2% to 11% patients who only have IMLN metastases but with no concurrent ALN metastases. Veronesi et al^[6]^ evaluated 1119 cases of breast cancer patients and found that 9.1% of them had positive IMLN but tumor-free of ALN. The study of Estourgie et al^[7]^ reached a similar conclusion: 7% of patients (9/130) has positive IMLN but negative ALN. In our earlier study, the rate of IMSLN metastasis was 8.1% in ALN-negative patients. The role of IMLN is important because of its impact on staging and accurate indication of radiation to the internal mammary area. Those patients who only had IMLN involvement would have an altered lymph node staging, which would lead to IMLN radiotherapy plan.^[8]^

With the continuous maturation of radiotherapy technology, internal mammary node irradiation (IMNI) which can improve the survival of breast cancer patients has gained more and more attention. The meta-analysis of 3 clinical trials-EORTC (n = 4004), MA.20 (n = 1832), and French trial (n = 1334) further confirmed that on the basis of the whole breast and the chest wall irradiation, additional IMNI and supraclavicular region radiotherapy could significantly improve the 10-year overall survival (HR = 0.88, 95%CI: 0.78–0.99) and 10-year disease-free survival (HR = 0.86, 95% CI:0.78–0.95).^[9]^ The 8-year follow-up results of DBCG-IMLN research showed that IMNI can significantly improve the overall survival (HR = 0.82, 95% CI:0.72–0.94, *P*<.005) and reduce the mortality (HR = 0.85, 95% CI:0.73–0.98, *P* = .03).^[10]^ The 2017 NCCN Guidelines recommended IMNI to patients with more than 4 positive ALNs (category 1), and strongly suggest IMNI to patients with 1 to 3 positive ALNs (category 2A). At present, the indication of IMNI mainly depends on the high-risk factors of IMLN metastases.^[11]^ However, patients with high-risk factors of IMLN metastases do not necessarily have IMLN metastases, and low-risk patients cannot be excluded of the possibility of IMLN metastases. Therefore, the inclusion criteria of the above studies (only patients with high-risk metastasis factors were selected without IMLN pathology assessment) can lead to a certain degree of under-/over-treatment. Taking the cardiac toxicity of IMNI into account, it is important to grasp the indications of IMNI.

The IMLN metastases is an independent prognostic factor for breast cancer patients.^[12]^ Patients with either IMLN or ALN metastases have the similar prognosis, while metastases of both of them indicates a very poor prognosis.^[13]^ The choice of adjuvant treatment regimens that rely solely on ALN staging is not sufficient, as patients have 2 main nodal regions (ALN and IMLN).^[9]^ According to the 8th edition of TNM classification, pathologically confirmed metastases only occurred in IMLN is classified as pN1b or pN2b.^[4]^ The combination of ALN and IMLN staging can provide a more reliable basis for pathological staging and adjuvant treatment regimens.

The above facts confirmed the necessity of IMLN detection, which suggest that we should always take the 2 possible lymphatic pathways into consideration in order to prevent neglecting the subgroup of patients with negative ALN and positive IMLN. A-SLNB has developed for precisely diagnose ALN metastases in breast cancer patients with clinically and iconography negative ALN. However, it is well known that IMLN usually has deep anatomical position and small diameter (<0.5 cm), the sensitivity of iconography methods cannot meet the clinical requirements to detect IMLN metastases,^[7]^ and extended radical mastectomy had been abandoned because it cannot improve the overall survival of patients.^[10]^ Therefore, we recommend IM-SLNB as the minimally invasive staging technique to assess the status of IMSLN, which can provide patients with more accurate staging to guide IMNI. Patients with positive IMSLN should receive IMNI, while IMNI could be avoided in those patients with negative IMSLN.^[13]^

However, IMSLN can be identified only in a small proportion of patients with the tradition injection technique, which has been the biggest restriction of IM-SLNB.^[1]^ IM-SLNB has not been performed routinely and has remained a subject of discussion. Based on the hypothesis that IMSLN receives lymphatic drainage not only from the primary tumor area, but also the entire breast parenchyma, Qiu et al^[3,8]^ injected radiotracer using modified injection technique (peri-areolar intra-parenchymal, high volume, and ultrasound guidance), which could significantly improve the preoperative visualization rate of the IMSLN (70.2%, 203/289). With the combination of the intraoperative gamma probe, the detection rate of IMSLN could reach 77.2% (223/289; *P*<.05). More patients could benefit from the detection of IMSLN. To verify the IMSLN hypothesis and modified injection technique, Cong injected 2 kinds of tracers (radiotracer and fluorescence tracer) at different sites of the breast intra-parenchyma: the radiotracer was injected with the modified injection technique, and the fluorescence tracer was injected in the peritumoral intra-parenchyma. He found that both of tracers could reach the same IMSLN.^[14,15]^ The accuracy of IMSLN hypothesis and modified injection technique had been demonstrated preliminarily from his study.

## 4 Conclusion

The clinical significance of positive IMSLN has not yet been precisely defined. Further large-scale case analysis will contribute to solve this clinical problem. The visualization rate of IMLN has been improved guided by modified injection technology. Up to now we can conclude that IM-SLNB is a safe and feasible minimally invasive diagnostic technique, which can help to determine the nodal staging and the pathological status of IMSLN in order to avoid under-staging and under-/over-treatment.
